# Biodiesel Production from Waste Cooking Oil Using Recombinant *Escherichia coli* Cells Immobilized into Fe_3_O_4_–Chitosan Magnetic Microspheres

**DOI:** 10.3390/molecules29153469

**Published:** 2024-07-24

**Authors:** Zexin Zhao, Meiling Han, Ling Zhou, Changgao Wang, Jianguo Lin, Xin Du, Jun Cai

**Affiliations:** Key Laboratory of Fermentation Engineering (Ministry of Education), Hubei Key Laboratory of Industrial Microbiology, Cooperative Innovation Center of Industrial Fermentation (Ministry of Education & Hubei Province), Hubei University of Technology, Wuhan 430068, China; zx_zhao@hbut.edu.cn (Z.Z.); 102200590@hbut.edu.cn (M.H.); 102210733@hbut.edu.cn (L.Z.); wcg20220908@163.com (C.W.); jianguolin@hbut.edu.cn (J.L.); xindu@hbut.edu.cn (X.D.)

**Keywords:** magnetic whole-cell catalyst, *Escherichia coli*, immobilization, waste cooking oil, biodiesel

## Abstract

Developing reusable and easy-to-operate biocatalysts is of significant interest in biodiesel production. Here, magnetic whole-cell catalysts constructed through immobilizing recombinant *Escherichia coli* cells (containing MAS1 lipase) into Fe_3_O_4_–chitosan magnetic microspheres (termed MWCC@MAS1) were used for fatty acid methyl ester (FAME) production from waste cooking oil (WCO). During the preparation process of immobilized cells, the effects of chitosan concentration and cell concentration on their activity and activity recovery were investigated. Optimal immobilization was achieved with 3% (*w*/*v*) chitosan solution and 10 mg wet cell/mL cell suspension. Magnetic immobilization endowed the whole-cell catalysts with superparamagnetism and improved their methanol tolerance, enhancing the recyclability of the biocatalysts. Additionally, we studied the effects of catalyst loading, water content, methanol content, and reaction temperature on FAME yield, optimizing these parameters using response surface methodology and Box–Behnken design. An experimental FAME yield of 89.19% was gained under the optimized conditions (3.9 wt% catalyst loading, 22.3% (*v*/*w*) water content, 23.0% (*v*/*w*) methanol content, and 32 °C) for 48 h. MWCC@MAS1 demonstrated superior recyclability compared to its whole-cell form, maintaining about 86% of its initial productivity after 10 cycles, whereas the whole-cell form lost nearly half after just five cycles. These results suggest that MWCC@MAS1 has great potential for the industrial production of biodiesel.

## 1. Introduction

With the depletion of petroleum fuel resources and the increasing awareness of reducing carbon footprint, the development of alternative liquid fuels from renewable and sustainable feedstocks has received significant attention. Biodiesel, primarily composed of fatty acid methyl esters (FAMEs), is a carbon-neutral and non-toxic substitute for petrodiesel that can be produced from a variety of feedstocks, such as vegetable oils, animal fats, microbial oils, and waste oils [1,2]. Currently, edible oils account for over 95% of global biodiesel production, inadvertently intensifying the food versus energy debate and inflating production costs [3]. An estimated 16.5 million tons of waste cooking oil (WCO) are produced annually worldwide, posing significant threats to the environment and human health due to improper treatment, such as illegal discharge and reuse of edible oil [4]. Utilizing WCO for biodiesel production offers a cost-effective (30–50% cheaper than vegetable oil), waste-minimizing, and environmentally friendly solution, thereby fostering a circular economy [5]. 

Transesterification of refined oil with methanol (i.e., methanolysis) catalyzed by alkaline catalysts is the most commonly used method in commercial biodiesel production. However, the chemically synthetic process is inappropriate for the conversion of WCO which usually contains a high amount of free fatty acids (FFAs), owing to the soap formation [6,7]. Despite the prepositive esterification of FFA with methanol/glycerol can depress the FFA of WCO to a range acceptable for alkaline treatment (below 1%), the use of acid catalysts and high energy consumption incurs additional financial and environmental costs [8,9,10]. In contrast, enzymatic methods, employing lipases to catalyze the conversion of both FFAs and glycerides into FAME, offer a greener alternative [11]. The process is generally conducted under mild conditions, reducing energy use and chemical corrosion, thus highlighting their environmental and sustainable advantages. However, the cost of enzymes remains a significant barrier.

Whole-cell catalysts (WCCs), microorganisms that produce lipase and maintain their cellular structure, are seen as viable biocatalysts for biodiesel production [12,13,14]. The main advantage of WCCs lies in their exemption from extraction and purification processes, substantially lowering production costs. In addition, the cellular environment can alleviate the inhibition of enzymes via substrates or products and keep them stable, though cellular structure may impede mass transfer. In our previous work, an *Escherichia coli* (*E. coli*) WCC was genetically engineered to express a non-regiospecific lipase from marine *Streptomyces* sp. W007 (namely, MAS1) and enabled high-yield production of biodiesel (>93%) [15,16,17]. Although the WCC exhibits the advantages of methanol resistance and reuse, the inability to easily recover remains changeling in its industrial application.

Immobilization generally facilitates WCC reusability and operational simplicity [18,19,20]. Especially, magnetic whole-cell catalysts (MWCCs) that are constructed by immobilizing cells on/in magnetic carriers can be easily separate from the reaction system with an external magnetic field [21,22,23]. Among them, the ferroferric oxide–chitosan (Fe_3_O_4_-CTS) microspheres emerge as exceptional candidates for the immobilization of microbial cells due to their facile accessibility, high biocompatibility, superparamagnetism, non-toxicity, and possession of plentiful functional groups [24,25]. In this work, MWCCs, constructed by immobilizing MAS1 lipase-producing *E. coli* into Fe_3_O_4_-CTS microspheres, were characterized and applied for the conversion of WCO into biodiesel. The optimization of immobilization and reaction parameters was conducted, and the optimum condition for maximum production was predicted via response surface methodology (RMS) and Box–Behnken design (BBD). Furthermore, the recyclability of the MWCC and its production capacity in scale-up reactions were also evaluated. 

## 2. Materials and Methods

### 2.1. Strains and Materials

Recombinant *E. coli* Rosetta-gamiB (DE3)-expressing MAS1 lipase was constructed in our previous work [15]. Antibiotic ampicillin (Amp), BSA standard protein, and isopropyl β-D-1-thiogalactopyranoside (IPTG) were purchased from Sangon (Shanghai, China). Chitosan (degree of deacetylation: 90%, Mw: 20000), 25% (*w/v*) glutaraldehyde aqueous solution, 2,7-dichlorofluorescein, and FAME standard (purity > 98%) were purchased from Macklin (Shanghai, China). A thirty-seven-component FAME qualitative mix was purchased from Merck KGaA (Darmstadt, Germany). WCO containing ca. 9 wt% FFA was obtained from local restaurants and centrifuged before use. Other reagents were of analytical grade and provided by a local supplier.

### 2.2. Synthesis of Fe_3_O_4_ Magnetic Nanoparticle

Magnetic iron oxide nanoparticles were synthesized via a modified chemical co-precipitation method described by Chen et al. [26] In brief, FeSO_4_·7H_2_O and FeCl_3_·6H_2_O were dissolved in a 1 wt% solution of polyethylene glycol at a molar ratio of 1:2. Subsequently, 25 wt% NH_3_·H_2_O solution was gradually added to the solution until precipitates formed. The resultant precipitates were collected using an external magnetic field and rinsed thoroughly with ddH_2_O. Finally, the collected precipitates were mixed with tetraethoxysilane, NH_3_·H_2_O, and ethanol to obtain SiO_2_-coated Fe_3_O_4_ magnetic nanoparticles. The final products were thoroughly rinsed with water and ethanol and then dried using a vacuum freeze dryer.

### 2.3. Immobilization of E. coil Cells in Fe_3_O_4_–Chitosan Magnetic Microspheres

The recombinant *E. coli* cells containing MAS1 lipase were prepared following the fermentation process described in our previous work [15]. The *E. coil* cells were collected from culture broth using centrifugation at 10,000× *g* and 4 °C for 15 min following a 21-h induction culture at 28 °C. The cells were then washed thoroughly with sodium phosphate buffer (0.1 M, pH 7.0) and resuspended in the wash buffer to a specific cell density. To prepare MWCC@MAS1, the cell suspension was thoroughly mixed with the chitosan solution (prepared by dissolving a certain mass of chitosan in 2% (*w/v*) acetic acid) containing 4% (*w/v*) magnetic nanoparticles with the aid of ultrasonic wave (1:12, *v*/*v*). The prepared mixture was added dropwise into sterilized 5% (*w/v*) NaOH solution using a sterile syringe. Subsequently, 25% (*w/v*) glutaraldehyde aqueous solution was added to solidify the formed beads with stirring for 2 h. The magnetic microspheres were then collected using an external magnetic field and washed repeatedly with ddH_2_O until a neutral pH was achieved. The activity recovery of the immobilized whole-cell catalysts was calculated using Equation (1):

(1) $$\mathrm{Activity}\;\mathrm{recovery}\;(\%)=\frac{A_{m}}{A_{c}}$$

where *A_c_* and *A_m_* (U) in Equation (1) are the total activities of the added cells and the prepared MWCCs, respectively.

### 2.4. Characterization of Magnetic Carrier and MWCC

The diameter of MWCCs was measured using a digital micrometer (San Liang, Dongguan, China). In order to observe the surface morphology of MWCCs, samples were lyophilized and covered with gold particles and then tested using a scanning electron microscope (SEM, Regulus 8100, Hitachi, Japan). The crystallographic structure of MWCCs was examined using X-ray diffraction (XRD) with a diffractometer (SmartLab SE, Rigaku, Japan) with Cu K_α1_ (λ = 1.5406 Å, 40 kV, 30 mA) radiation-, and the scanning rate was 5°/min. The magnetization curve of samples was recorded using a vibrating sample magnetometer (VSM, LakeShore 8604, Lexington, MI, America).

### 2.5. Lipase Activity Assay

The lipase activity of WCCs and MWCCs was measured using the olive oil emulsion method described previously [15]. The reaction mixture consisted of 5 mL of reaction buffer (0.1 M, pH 7.5 sodium phosphate buffer), 4 g of emulsified oil (olive oil: polyvinyl alcohol = 1:3, *v*/*v*), and 0.1 g of MWCCs (or equivalent WCCs). This mixture was incubated in a thermostatic shaking water bath at 300 rpm and 40 °C for 15 min. To terminate the reactions, 15 mL of 95% ethanol was added, followed by titration of the released fatty acids with 0.05 M KOH. One unit of activity (U) was defined as the amount of catalyst required to generate 1 μmol of free fatty acid per minute. In control experiments, heat-inactivated biocatalysts were used instead of their active state.

### 2.6. Methanol Resistance Assays

The determination of the methanol tolerance of MWCC@MAS1 was performed by incubating the catalyst in 30% and 50% methanol solution (diluted with reaction buffer) at 30 °C for a certain time, respectively. An external magnetic field was used to separate the MWCCs from the methanol solution, and their residual hydrolytic activity was then measured immediately using the method mentioned above. 

### 2.7. MWCC-Catalyzed Synthesis of FAME

The synthesis of FAMA catalyzed by MWCC@MAS1 was conducted in a 20 mL transparent plastic bottle with magnetic stirring at 400 rpm. The optimization of the reaction conditions was implemented using a single-factor experiment. The effects of catalyst loading (1–5 wt%), water content (15–50% (*v*/*w*)), methanol content (20–35% (*v*/*w*)), and temperature (24–36 °C) on FAME yield were systematically investigated. The initial conditions were set as 2 g of WCO, 30% (*v*/*w*) reaction buffer, 30% (*v*/*w*) methanol (ca. 2.7 molar equivalents based on acyl groups), and 30 °C. Thereafter, RSM coupled with BBD was employed to predict the optimal conditions for biodiesel synthesis from WCO. Finally, a 100-fold scale-up reaction (containing 200 g of WCO) was carried out in a 1 L flask under the optimal conditions determined. Samples were taken at 6, 12, 24, 36, 48, 60, and 72 h during catalyst loading optimization, and withdrawn at 48 h in other reaction systems.

### 2.8. Components Analysis of Reaction Mixtures

The lipid classes of WCO and reaction mixtures were analyzed using the high-performance liquid chromatography (HPLC) method described in our previous work [15]. Briefly, samples dissolved in the mobile phase (*n*-hexane: 2-propanol: methanoic acid = 13:1:0.003, *v*/*v*/*v*) were analyzed using a Shimadzu LC-20AD HPLC system equipped with an RID-10A detector and a SiO_2_ column (Welch, Shanghai, China, 4.6 × 250 mm). This setup was used to detect the contents of triacylglycerol (TAG), diacylglycerol (DAG), monoacylglycerol (MAG), free fatty acids (FFAs), and FAME. The FAME yield in this study was calculated as follows (Equation (2)):

(2) $$\mathrm{FAME}\;\mathrm{yield}\;(\%)=\;\frac{FAME(wt\%)}{TAG\left(wt\%\right)+DAG\left(wt\%\right)+MAG\left(wt\%\right)+FFA\left(wt\%\right)+FAME(wt\%)}\times 100\%$$

The FA composition of WCO and the biodiesel product was analyzed following a modified method described by Wang et al. [27]. The WCO was converted to FAMEs following the boron trifluoride methylation procedure [28], and the products were extracted with *n*-hexane. With regard to biodiesel products, the separation of FAMEs was applied to thin layer chromatography (TLC) plates (10 × 20 cm) coated with silica gel (HSGF254, Jiangyou silica gel, Yantai, China). The TLC plates were developed with *n*-hexane/dichloromethane/ethyl acetate/acetic acid (16:3.5:0.3:0.2, *v/v/v/v*). After co-chromatography with the FAME standard, the bands of lipid classes were stained with 0.2% (*w/v*) 2,7-dichlorofluorescein in methanol. The FAME band was scraped off under UV light and dissolved in *n*-hexane. The samples prepared from WCO and the reaction mixture were then analyzed using a Shimadzu GC 2030-FID system equipped with a CP-Sil 88 column (60 m  ×  0.25 mm  ×  0.2 µm, Agilent Technologies) with nitrogen as the carrier gas (at a head pressure of 179.6 kPa). The injection of detector temperatures was set at 200 and 220 °C, respectively. The column oven was initially held at 140 °C for 5 min, then increased to 200 °C at a rate of 4 °C/min and held isothermally for 2 min, and finally raised to 220 °C at a rate of 4 °C/min and held isothermally for 13 min. The content of each substance was determined by the area normalization method.

### 2.9. Recycling of MWCC for FAME Production

A mixture containing 2 g of WCO, 78 mg of MWCC@MAS1, 446 μL of reaction buffer, and 460 μL of methanol was stirred at 32 °C and 400 rpm for 48 h. A magnet was applied to separate the magnetic catalysts from the mixture. The liquid portion was centrifuged at 10,000× *g* and 4 °C for 10 min, and the upper phase was analyzed using HPLC. The catalyst was washed sequentially with *n*-hexane and reaction buffer twice then dried and mixed with fresh substrates to start the next cycle. To compare the reusability of MWCC@MAS1 with its WCC form (termed WCC@MAS1), biodiesel production using WCC@MAS1 was also conducted. The reaction conditions for the WCC-catalyzed system included 2 g of WCO, 1.5 wt% WCC@MAS1, 1.2 mL of 50% (*v*/*v*) methanol, 29 °C, and 400 rpm. The recycling process for WCCs followed the method described in our previous study [15].

### 2.10. Statistical Analysis

All experiments were performed in triplicate. All results in the present study are presented as the means ± standard deviations (SD).

## 3. Results and Discussion

### 3.1. Effect of Immobilization Parameters on Lipase Activity of MWCC@MAS1

**Figure 1:**
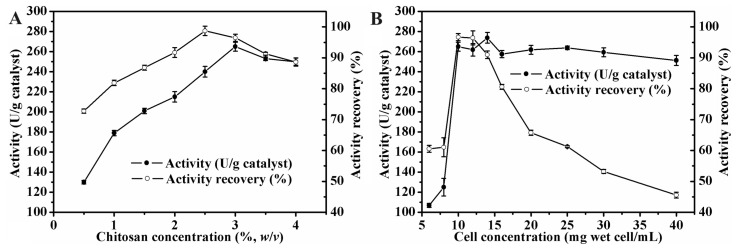
Optimization of the process of immobilizing recombinant *E. coli* cells in magnetic chitosan microspheres: (**A**) Effect of chitosan concentration on lipase activity and activity recovery of MWCC@MAS1. (**B**) Effect of cell concentration on lipase activity and activity recovery of MWCC@MAS1.

The immobilization of recombinant *E. coli* cells producing the MAS1 lipase in magnetic Fe_3_O_4_-CTS microspheres was explored to construct a new MWCC system. As shown in Figure 1A, with an added cell suspension concentration of 10 mg wet cell/mL, the activity of MWCC@MAS1 increased significantly as the chitosan concentration was raised from 0.5% to 3% (*w/v*), reaching a maximum lipase activity of 265.0 U/g for the catalyst. However, further increases in chitosan concentration led to a decrease in activity. Additionally, the activity recovery of the immobilization process exceeded 95% when the chitosan concentration ranged from 2.5% to 3% (*w/v*). These changes could be attributed to excessively high concentrations of chitosan, which may cause the microspheres to become overly compact, thereby affecting substrate mass transfer.

Next, the effect of the cell loading amount on the activity of MWCC@MAS1 was investigated (Figure 1B). Before the concentration of the added cell suspension reached 10 mg wet cell/mL, the activity of the MWCCs increased as the cell concentration increased. However, beyond this concentration, further increases did not significantly influence catalyst activity, and their activity recovery began to decline sharply. Excessive cell loading led to cell stacking of cells within the microspheres, blocking contact between cells and substrates, thereby reducing activity recovery rates. Considering cost and activity, the optimum immobilization parameters for MWCC@MAS1 were identified as 3% (*w/v*) chitosan concentration and 10 mg wet cell/mL cell concentration.

### 3.2. Characterization of MWCC@MAS1

**Figure 2:**
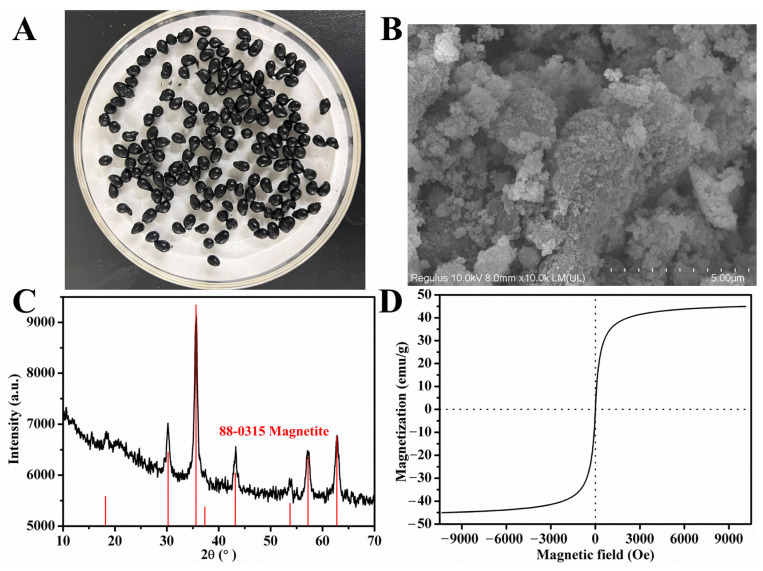
Characterization of the developed MWCC@MAS1 system: (**A**) Appearance of the magnetic particles. (**B**) Micromorphology of MWCC@MAS1 obversed with SEM. (**C**) Comparison of the XRD patterns between MWCC@MAS1 (black line) and Fe_3_O_4_ (red line). (**D**) Magnetization curves of MWCC@MAS1 at room temperature.

The morphology of the prepared MWCCs is shown in Figure 2A. The particles exhibit a black and near-spherical appearance with average diameters ranging from 2 to 5 mm. The micro-morphology of MWCCs was examined using SEM (Figure 2B), revealing that the magnetic nanoparticles and *E. coli* cells were constrained by cross-linked chitosan, resulting in a grainy surface of the microspheres. Furthermore, a porous structure was observed, facilitating the mass transfer of substrates.

**Figure 3:**
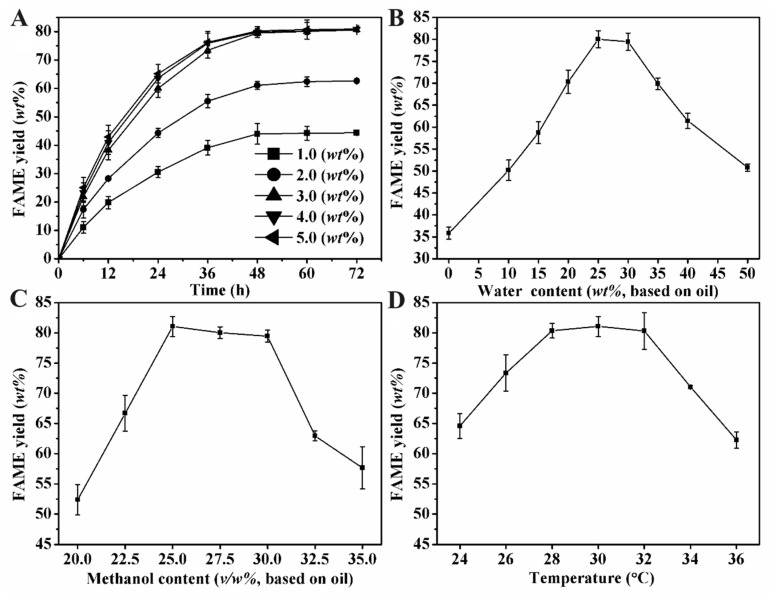
Single factor optimization of biodiesel production conditions catalyzed by MWCC@MAS1: (**A**) Effect of catalyst loading on the reaction equilibrium. Reaction systems contained 2 g WCO, 30% (*v*/*w*) water content, 30% (*v*/*w*) methanol content, and a certain amount of MWCC@MAS1. (**B**) Effect of water content on FAME yield. Reaction systems contained 2 g WCO, 3 wt% magnetic catalysts, 30% (*v*/*w*) methanol content, and a certain volume of reaction buffer. (**C**) Effect of methanol content on FAME yield. Reaction systems contained 2 g WCO, 25% (*v*/*w*) water content, 3 wt% magnetic catalysts, and a certain volume of methanol. Reactions mentioned in (**A**–**C**) were carried out at 30 °C and 400 rpm magnetic stirring, and the reaction time of (**B**,**C**) was 48 h. (**D**) Effect of temperature on FAME yield. Reaction systems contained 2 g WCO, 3 wt% magnetic catalysts, 25% (*v*/*w*) water content, and 25% (*v*/*w*) methanol content, and the reactions were performed at a certain temperature and 400 rpm stirring for 48 h.

The XRD pattern of MWCCs (Figure 3C) displayed the peaks at 2θ = 30.3°, 35.6°, 43.3°, 53.7°, 57.2°, and 62.9°, corresponding to the (220), (311), (400), (422), (511), and (440) crystallographic planes of Fe_3_O_4_, respectively (JCPDS card 88-0315, its standard spectrum shown by red lines in Figure 3C) [29]. The peak at 2θ = 20.0° related to chitosan was relatively weak, confirming the amorphous form of chitosan in MWCCs [30]. The mean diameter of *E. coli* cells immobilized Fe_3_O_4_, calculated using the Scherrer equation, is approximately 12.2 nm, which is inferior to the superparamagnetic critical size (20 nm) [31]. In addition, the magnetic property of MWCCs was evaluated using VSM at room temperature. As shown in Figure 3D, the magnetic microspheres exhibited an S-shaped magnetization curve with a saturation magnetization of 44.9 emu/g and negligible coercivity and remanence. These results indicated that the prepared MWCCs exhibit superparamagnetic behavior [32].

The excellent methanol tolerance of WCC@MAS1 had been well demonstrated in our previous work [15]. In this study, the effect of magnetic immobilization on the methanol tolerance of the WCCs was investigated. As shown in Table 1, the deactivation half-lives of MWCC@MAS1 in 30% and 50% (*v*/*v*) methanol solutions ($t_{1/2}^{M,30\%}$ and $t_{1/2}^{M,50\%}$) were both slightly increased compared with those of WCC@MAS1 (42.76% in 30% methanol, and 38.53% in 50% methanol). This may be attributed to the protective effect of the chitosan shell. The polar groups in chitosan interacted with methanol molecules via hydrogen bonds, limiting the diffusion of methanol from solvent to cell, thereby alleviating enzyme deactivation. 

### 3.3. Effects of Synthesis Conditions on Biodiesel Yield

Given the considerable methanol tolerance of MWCC@MAS1, a one-step methanol addition method was implemented for biodiesel production from WCO in this study. As shown in Figure 3A, the time course of FAME accumulation at 30 °C under the conditions of 30% (*v*/*w*, based on WCO) reaction buffer and 30% (*v*/*w*) methanol (ca. 2.7 molar equivalents based on WCO acyl groups), indicates that all reaction systems reached equilibrium at 48 h. The rate of FAME accumulation increased substantially with an increase in MWCC@MAS1 loading from 1 to 3 wt% (based on WCO). About 80% FAME yield was obtained at a catalyst content of 3 wt% after 48 h of reaction. Further increases in catalyst content did not significantly improve the accumulation rate and yield. Compared to the MWCC system with lipase-producing *Pseudomonas mendocina*, which required more than 10 wt% catalyst loading with four-step methanol addition [26], only 3 wt% MWCC@MAS1 was sufficient to reach a similar FAME yield. Its catalyst consumption (~0.03 g_MWCC@MAS1_/g_FAME_) is even lower than that of other WCCs [33,34] and comparable to some chemical catalysts [35,36], indicating the great potential of MWCC@MAS1 for industrial application. Under the same conditions, when magnetic chitosan microspheres without cells were added to the system instead of MWCC@MAS1, no FAME products were detected after 48 h. This indicates that the catalytic ability of the magnetic catalyst is provided by the recombinant *E. coli* cells.

Water content is another critical factor influencing biodiesel yield in biocatalysis. As shown in Figure 3B, the FAME yield increased sharply with an increase in water content from 0 to 25 wt%, reaching a maximum yield of 80.06% at 25 wt%. The result indicates that adding a moderate amount of water can alleviate methanol inactivation and enhance the interfacial area, thereby activating lipase transesterification activity and promoting product accumulation [37]. However, further increase in water content led to a decrease in FAME yield due to the increased hydrolysis activity of lipase in excess water [15,38].

Methanol, a molecule with dual functions (reaction substrate and lipase inactivator), is closely related to product yield in enzymatic biodiesel preparation from WCO. As shown in Figure 3C, the FAME yield increased with an increase in methanol content from 20 to 25 wt%, reaching a maximum yield of 81.08% at 25 wt% methanol (Figure 3C). Increasing the methanol content to 30 wt% slightly decreased the biodiesel yield to 79.47%, and a dramatic drop to below 65% was observed with further increases to 32.5 wt%. The results demonstrated that MWCC@MAS1 activity is inhibited by higher methanol concentration (over 50% (*v*/*v*)), consistent with its methanol inactivation experiments.

The effect of temperature on the FAME yield catalyzed using MWCC@MAS1 was also explored, as depicted in Figure 3D. Increasing the temperature from 24 to 30 °C enhanced FAME accumulation. However, further increases beyond 32 °C significantly reduced the yield. Higher temperatures increase the kinetic energy within the reaction system, enhancing mass transfer and promoting efficient collisions between the catalyst and substrates. Conversely, excessively high temperatures can activate cellular processes that lead to lipase degradation, consequently diminishing biodiesel production. Similar observations have been reported in other studies [21,26].

### 3.4. Optimization of Reaction Parameters for Biodiesel Production

The RSM technique, implemented through Design-Expert software (version 9), was employed to optimize the biodiesel synthesis parameters, using MWCC@MAS1 as the catalyst. This optimization utilized a three-level-four-factor BBD, comprising 27 experimental runs to fit a second-order response surface (the codes of the selected variables and their levels are shown in Table 2). The design matrix, which includes both predicted and actual values from each experimental run, is displayed in Table 3. Subsequent analysis of the experimental data via BBD led to the formulation of a second-order polynomial equation (Equation (3)), elucidating the mathematical relationship between the variables and the yield of biodiesel.

Y_(yield)_ = 81.13 + 1.65A − 4.63B − 3.65C + 2.01D − 0.815AB + 2.06AC + 2.77AD + 0.6025BC + 0.94BD − 2.38CD  − 2.65A^2^ − 4.34B^2^ − 3.21C^2^ − 1.25D^2^ (3)

The fitness and significance of the regression model were assessed through an analysis of variance (ANOVA). As shown in Table 4, the *F*-value and *p*-value of the model were 55.13 and less than 0.0001, respectively, suggesting the high statistical significance of the predicted model [39,40,41]. The determination coefficient (R^2^ = 0.9848) implied that only 1.52% of the total variation was not explained by the model. The adjusted determination coefficient (R_Adj_^2^ = 0.9668) revealed a slight difference between predicted and experimental values [42,43]. The results demonstrated that the regression model was reliable and precise in reflecting the mutual effect of variables and predicting the response values. 

**Figure 4:**
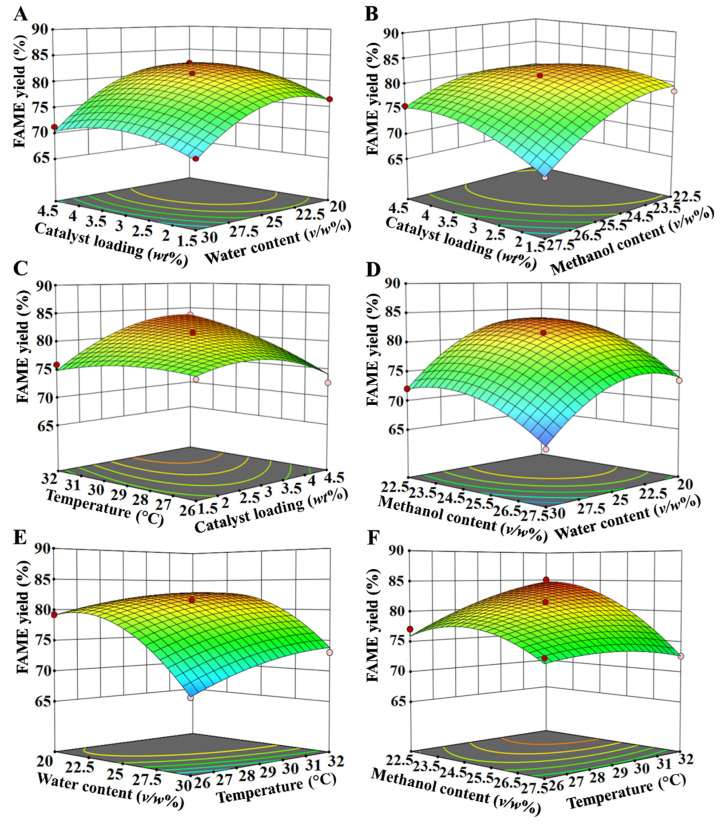
Three-dimensional response surface plots of variables influencing FAME yield from WCO: (**A**) Effect of catalyst loading and water content on FAME yield. (**B**) Effect of catalyst loading and methanol content on FAME yield. (**C**) Effect of temperature and catalyst loading on FAME yield. (**D**) Effect of methanol content and water content on FAME yield. (**E**) Effect of water content and temperature on FAME yield. (**F**) Effect of methanol content and temperature on FAME yield.

The 3D response surfaces were plotted to further illustrate the reciprocal influences on the MWCC-catalyzed biodiesel yield (Figure 4). Based on the mathematic model, a maximum biodiesel yield of 86.62% is predicted under the conditions of 3.9 wt% catalyst loading, 22.3% (*v*/*w*) water content, 23.0% (*v*/*w*) methanol content, and 32 °C. This predicted value was close to the experimentally determined biodiesel yield of 89.19% under these conditions, suggesting the accuracy of the regression model.

Based on the optimization study results, a 100-fold scale-up of biodiesel production was carried out in a 5 L flask containing 200 g of WCO. The FAME yield of the reaction system at 48 h was 87.36%, closely mirroring the semi-preparative reaction and demonstrating good reproducibility of the biodiesel production process across different scales.

### 3.5. FA Composition Analysis of FAME

**Figure 5:**
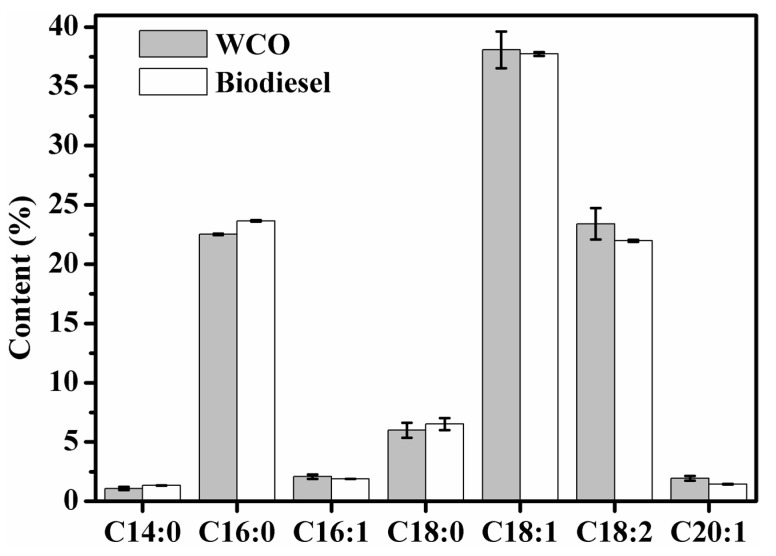
Fatty acid composition analysis of WCO and biodiesel product.

The FA preference of biocatalyst toward glyceride also influences the conversion efficiency of WCO to FAME [44]. As shown in Figure 5, the FA composition of the final FAME product in semi-preparative system resembled that of the WCO substrate, consisting primarily of 23.65% palmitic acid (C16:0), 6.51% stearic acid (C18:0), 37.74% oleic acid (C18:1), and 21.98% linoleic acid (C18:2). The results indicated that MWCC@MAS1 presents a weak preference for the acyl donors in the substrate, which is consistent with the fatty acid selectivity of MAS1 lipase [45].

### 3.6. Recycling of MWCC@MAS1 in the Conversion of WCO to Biodiesel

**Figure 6:**
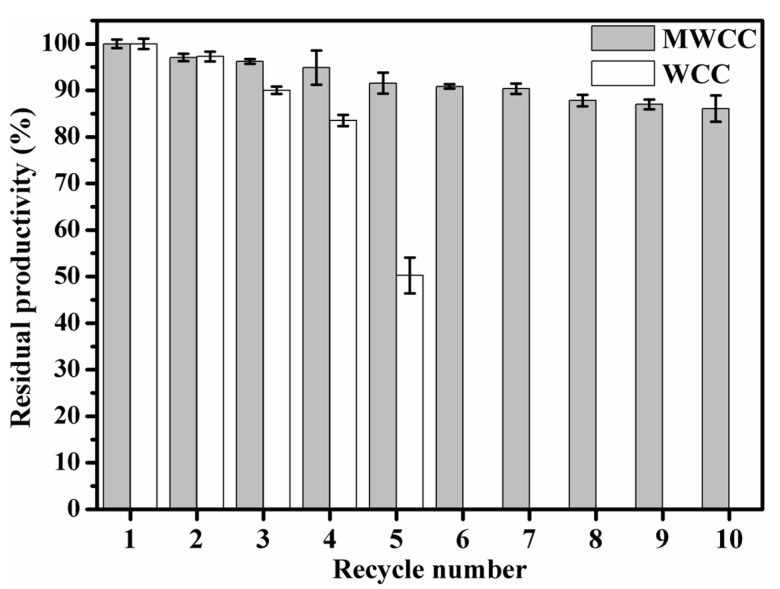
Recycling of the WCC@MAS1 and MWCC@MAS1 in the conversion of WCO into FAME. The WCC-catalyzed reaction system contained 2 g WCO, 1.5 wt% WCC@MAS1, 30% (*v*/*w*) reaction buffer and 30% (*v*/*w*) methanol. The reactions were performed at 29 °C and 400 rpm magnetic stirring for 48 h. T WCC-catalyzed reaction system contained 2 g WCO, 3.9 wt% catalyst loading, 22.3% (*v*/*w*) water content, 23.0% (*v*/*w*) methanol content, and 32 °C) were used in MWCC-catalyzed reaction system. The FAME yield in the first cycle was set as 100% productivity.

The reusability of MWCC@MAS1 was explored over 10 cycles of biodiesel preparation under the optimized conditions and compared with that of its WCC form. As illustrated in Figure 6, MWCC@MAS1 maintained about 86% of its initial productivity after the tenth reuse, while a remarked decline in biodiesel yield catalyzed by WCC@MAS1 was observed after the fifth cycle, retaining only 50.25% productivity. The magnetic catalyst demonstrates superior reusability compared to other magnetic catalysts like *Candida antarctica* lipase B (CALB) immobilized on magnetic titanium graphene oxide (retaining 18.7% productivity after the ninth cycle) [46], lipases from *Aspergillus oryzae* and *Rhizomucor miehei* co-immobilized in magnetic chitosan microcapsules (retaining >50% productivity after the fifth cycle) [47], and CALB and *Aspergillus oryzae* lipase co-immobilized on Co^2+^-chelated magnetic metal–organic frameworks (retaining about 80% productivity after the tenth cycle) [48]. This enhanced reusability is crucial for efficient catalytic processes, potentially reducing costs and environmental impact by minimizing the need for frequent catalyst replacement. The cellular structure of WCCs could be disrupted by shear force generated by string and extrusion by centrifugation, leading to leakage of intracellular lipase and a decrease in productivity. Magnetically immobilized cells are protected by carriers and can be easily separated from the reaction system using an external magnetic field, resulting in less cellular structure damage and greater activity retention. Using a uniform axial magnetic field instead of mechanical agitation can reduce collisions between MWCC particles, thus further prolonging their service life. Based on the considerable superparamagnetism and durability, the magnetic catalysts show great application potential for constructing magnetically fluidized bed reactors to achieve continuous biodiesel production [49].

## 4. Conclusions

A novel biocatalyst constructed by immobilizing recombinant *E. coli* cells into Fe_3_O_4_–chitosan magnetic microspheres was successfully applied for the transformation of WCO into biodiesel. The methanol tolerance of the MWCCs is slightly better than that of their WCC form, making them suitable for the one-step methanol addition method. A biodiesel yield of 89.19% was achieved under optimal reaction conditions (3.9 wt% catalyst loading, 22.3% (*v*/*w*) water content, 23.0% (*v*/*w*) methanol content, and 32 °C) over 48 h. This yield was well reproduced in a 100-fold scale-up system. Moreover, the MWCCs demonstrated superior reusability compared to the corresponding WCCs, retaining more than 86% productivity after ten preparations. Therefore, the MWCCs developed here offer a promising approach to the industrial preparation of biodiesel. Different immobilization strategies and carriers significantly impact the performance of MWCCs. Continuous exploration and development of high-performance MWCCs could promote their industrial application. Additionally, incorporating MWCCs into columns to develop magnetically fluidized bed reactors capable of continuous production is another method to further enhance the cost-effectiveness of the catalysts.

## Figures and Tables

**Table 1:** The half-lives of WCC@MAS1 and MWCC@MAS1 were incubated in different concentrations of methanol.

	WCC@MAS1	MWCC@MAS1
$t_{1/2}^{M,30\%}\;(h)$	51.01 ± 1.23 [15]	72.82 ± 0.58
$t_{1/2}^{M,50\%}\;(h)$	6.80 ± 0.01 [15]	9.42 ± 0.32

**Table 2:** Matrix of experiments and the levels of the selected variables.

Variables	Code	Levels		
		−1	0	1
Catalyst loading (wt%)	A	30	60	90
Water content (*v*/*w*%)	B	400	500	600
Methanol content (*v*/*w*%)	C	450	500	550
Temperature (°C)	D	26	29	32

**Table 3:** Experimental and RSM model biodiesel yields.

Run	Coded Value of Reaction Variables				FAME Yield (%)	
	A	B	C	D	Predicted	Experimental
1	−1	−1	0	0	76.32	76.53
2	1	−1	0	0	81.26	82.07
3	−1	1	0	0	68.68	69
4	1	1	0	0	70.34	71.28
5	0	0	−1	−1	75.93	77.1
6	0	0	1	−1	73.39	74.19
7	0	0	−1	1	84.71	85.06
8	0	0	1	1	72.65	72.63
9	−1	0	0	−1	76.35	75.89
10	1	0	0	−1	74.11	72.56
11	−1	0	0	1	74.83	75.89
12	1	0	0	1	83.67	83.64
13	0	−1	−1	0	82.74	82.43
14	0	1	0	0	72.15	72.06
15	0	−1	1	0	73.97	73.41
16	0	1	1	0	65.89	65.45
17	−1	0	−1	0	79.34	78.31
18	1	0	−1	0	78.52	77.95
19	−1	0	1	0	67.92	67.8
20	1	0	1	0	75.34	75.68
21	0	−1	0	−1	79.11	79.23
22	0	1	0	−1	67.95	67.87
23	0	−1	0	1	81.25	80.65
24	0	1	0	1	73.85	73.05
25	0	0	0	0	81.13	81.01
26	0	0	0	0	81.13	81.58
27	0	0	0	0	81.13	80.8

**Table 4:** Analysis of variance for the RSM model.

Source	Sum of Squares	*df*	Mean Squares	*F*-Value	*p*-Value	
Model	703.07	14	50.22	55.13	< 0.0001	significant
Residual	10.93	12	0.9109	-	-	-
Lack of Fit	10.61	10	1.06	6.51	0.1404	not significant
Pure Error	0.3258	2	0.1629	-	-	-
Cor Total	714	26	-	-	-	-
R^2^ = 0.9848; R_Adj_^2^ = 0.9668						

## Data Availability

The original contributions presented in the study are included in the article, further inquiries can be directed to the corresponding author.

## References

[1] Bashir M.A., Wu S., Zhu J., Krosuri A., Khan M.U., Ndeddy Aka R.J. (2022). Recent development of advanced processing technologies for biodiesel production: A critical review. Fuel Process. Technol..

[2] Topare N.S., Jogdand R.I., Shinde H.P., More R.S., Khan A., Asiri A.M. (2022). A short review on approach for biodiesel production: Feedstock’s, properties, process parameters and environmental sustainability. Mater. Today Proc..

[3] Mathew G.M., Raina D., Narisetty V., Kumar V., Saran S., Pugazhendi A., Sindhu R., Pandey A., Binod P. (2021). Recent advances in biodiesel production: Challenges and solutions. Sci. Total Environ..

[4] Rocha-Meneses L., Hari A., Inayat A., Yousef L.A., Alarab S., Abdallah M., Shanableh A., Ghenai C., Shanmugam S., Kikas T. (2023). Recent advances on biodiesel production from waste cooking oil (WCO): A review of reactors, catalysts, and optimization techniques impacting the production. Fuel.

[5] Hosseinzadeh-Bandbafha H., Nizami A.-S., Kalogirou S.A., Gupta V.K., Park Y.-K., Fallahi A., Sulaiman A., Ranjbari M., Rahnama H., Aghbashlo M. (2022). Environmental life cycle assessment of biodiesel production from waste cooking oil: A systematic review. Renew. Sustain. Energy Rev..

[6] Atadashi I.M., Aroua M.K., Abdul Aziz A.R., Sulaiman N.M.N. (2012). Production of biodiesel using high free fatty acid feedstocks. Renew. Sustain. Energy Rev..

[7] Keera S.T., El Sabagh S.M., Taman A.R. (2011). Transesterification of vegetable oil to biodiesel fuel using alkaline catalyst. Fuel.

[8] Yeom S.H., Go Y.W. (2018). Optimization of a novel two-step process comprising re-esterification and transesterification in a single reactor for biodiesel production using waste cooking oil. Biotechnol. Bioprocess Eng..

[9] Dos Santos L.K., Hatanaka R.R., de Oliveira J.E., Flumignan D.L. (2017). Experimental factorial design on hydroesterification of waste cooking oil by subcritical conditions for biodiesel production. Renew. Energy.

[10] Elgharbawy A.S., Sadik W.A., Sadek O.M., Kasaby M.A. (2021). Maximizing biodiesel production from high free fatty acids feedstocks through glycerolysis treatment. Biomass Bioenergy.

[11] Cavalcante F.T.T., Neto F.S., Rafael de Aguiar Falcão I., Erick da Silva Souza J., de Moura Junior L.S., da Silva Sousa P., Rocha T.G., de Sousa I.G., de Lima Gomes P.H., de Souza M.C.M. (2021). Opportunities for improving biodiesel production via lipase catalysis. Fuel.

[12] Madavi T.B., Chauhan S., Keshri A., Alavilli H., Choi K.-Y., Pamidimarri S.D.V.N. (2022). Whole-cell biocatalysis: Advancements toward the biosynthesis of fuels. Biofuels Bioprod. Biorefin..

[13] Yaashikaa P.R., Kumar P.S., Karishma S. (2022). Bio-derived catalysts for production of biodiesel: A review on feedstock, oil extraction methodologies, reactors and lifecycle assessment of biodiesel. Fuel.

[14] Ye M., Ye Y., Du Z., Chen G. (2021). Cell-surface engineering of yeasts for whole-cell biocatalysts. Bioprocess Biosyst. Eng..

[15] Zhao Z., Huang J., Xu L., Wang C., Cai J. (2023). One-step production of biodiesel by wet Escherichia coli cells expressing a non-specific and methanol-resistant lipase. Process Biochem..

[16] Zhao Z., Chen S., Xu L., Cai J., Wang J., Wang Y. (2022). Structural basis for the regiospecificity of a lipase from *Streptomyces* sp. W007. Int. J. Mol. Sci..

[17] Zhao Z., Hou S., Lan D., Wang X., Liu J., Khan F.I., Wang Y. (2017). Crystal structure of a lipase from *Streptomyces* sp. strain W007—Implications for thermostability and regiospecificity. FEBS J..

[18] Lapponi M.J., Méndez M.B., Trelles J.A., Rivero C.W. (2022). Cell immobilization strategies for biotransformations. Curr. Opin. Green Sustain. Chem..

[19] Su Y., Li Q., Gu S., Liu Q., He W., Huang J., Wu W., Qi F. (2023). Development of biochar-based whole-cell biocatalysts for the production of L-tryptophan and L-phenylalanine. ACS Sustain. Chem. Eng..

[20] Pan T., Wang Z. (2023). Microbial biocatalysis. Catalysts.

[21] Liu J., Chen G., Yan B., Yi W., Yao J. (2022). Biodiesel production in a magnetically fluidized bed reactor using whole-cell biocatalysts immobilized within ferroferric oxide-polyvinyl alcohol composite beads. Bioresour. Technol..

[22] Chen G., Liu J., Yao J., Qi Y., Yan B. (2017). Biodiesel production from waste cooking oil in a magnetically fluidized bed reactor using whole-cell biocatalysts. Energy Convers. Manag..

[23] Tohfegar E., Habibi A. (2023). Immobilization of Candida catenulata cells by surface-loading of an amino-functionalized Fe_3_O_4_ nanoparticles and its application as the sustainable whole-cell biocatalyst for enzymatic biodiesel production. Energy Convers. Manag..

[24] Zhang C., Liu S., Li S., Tao Y., Wang P., Ma X., Chen L. (2019). Enahanced biosorption of Cu(II) by magnetic chitosan microspheres immobilized Aspergillus sydowii (MCMAs) from aqueous solution. Colloids Surf. A Physicochem. Eng. Asp..

[25] Negi H., Verma P., Singh R.K. (2021). A comprehensive review on the applications of functionalized chitosan in petroleum industry. Carbohydr. Polym..

[26] Chen G., Liu J., Qi Y., Yao J., Yan B. (2016). Biodiesel production using magnetic whole-cell biocatalysts by immobilization of Pseudomonas mendocina on Fe_3_O_4_-chitosan microspheres. Biochem. Eng. J..

[27] Wang X., Qin X., Li D., Yang B., Wang Y. (2017). One-step synthesis of high-yield biodiesel from waste cooking oils by a novel and highly methanol-tolerant immobilized lipase. Bioresour. Technol..

[28] Wang Y.-H., Mai Q.-Y., Qin X.-L., Yang B., Wang Z.-L., Chen H.-T. (2010). Establishment of an evaluation model for human milk fat substitutes. J. Agric. Food Chem..

[29] Xu J., Zeng G., Lin Q., Gu Y., Wang X., Feng Z., Sengupta A. (2022). Application of 3D magnetic nanocomposites: MXene-supported Fe_3_O_4_@CS nanospheres for highly efficient adsorption and separation of dyes. Sci. Total Environ..

[30] Yang W., Liu Y., Zhu Y., Jiang W., Shi F., Hu J., Jiang S., Jian S. (2023). Epichlorohydrin and triethylenetetramine functionalized electrosprayed Fe_3_O_4_/Chitosan magnetic microspheres for removal and separation of Congo red. Chem. Eng. J..

[31] Li L., Jiang W., Luo K., Song H., Lan F., Wu Y., Gu Z. (2013). Superparamagnetic iron oxide nanoparticles as MRI contrast agents for non-invasive stem cell labeling and tracking. Theranostics.

[32] Zhang Y., Wang D., Bai X., Xu J., Zhang J., Zhang G., Huang C., Liu W., Huang C., Xiong X. (2023). Microfluidic preparation of magnetic chitosan microsphere and its adsorption towards Congo red. J. Polym. Res..

[33] Tian K., Li Z. (2016). High-yielding, one-pot, and green production of biodiesel from waste grease using wet cells of a recombinant Escherichia coli strain as catalyst. Biochem. Eng. J..

[34] Yan J., Li A., Xu Y., Ngo T.P.N., Phua S., Li Z. (2012). Efficient production of biodiesel from waste grease: One-pot esterification and transesterification with tandem lipases. Bioresour. Technol..

[35] Sudalai S., Vairaprakash P., Devanesan M.G., Arumugam A. (2023). Sustainable biodiesel production from Madhuca indica oil using a functionalized industrial waste as a catalyst: Ready to scale-up approach. Ind. Crop. Prod..

[36] Karkal S.S., Rathod D.R., Jamadar A.S., Mamatha S.S., Kudre T.G. (2023). Production optimization, scale-up, and characterization of biodiesel from marine fishmeal plant oil using Portunus sanguinolentus crab shell derived heterogeneous catalyst. Biocatal. Agric. Biotechnol..

[37] Abdulla R., Derman E., K. Mathialagan T., Yaser A.Z., Abu Samah M.A., Gansau J.A., Syed Najmuddin S.U.F. (2022). Biodiesel production from waste palm cooking oil using immobilized *Candida rugosa* lipase. Sustainability.

[38] Karunanithi G., Varadappan A.M.S. (2023). Enzymatic transesterification of Phoenix dactylifera L. oil using lipase immobilized on activated carbon and optimization using response surface methodology. Biomass Convers. Biorefin..

[39] Dharmalingam B., Balamurugan S., Wetwatana U., Tongnan V., Sekhar C., Paramasivam B., Cheenkachorn K., Tawai A., Sriariyanun M. (2023). Comparison of neural network and response surface methodology techniques on optimization of biodiesel production from mixed waste cooking oil using heterogeneous biocatalyst. Fuel.

[40] Gonçalves M.A., dos Santos H.C.L., da Silva M.A.R., da Cas Viegas A., da Rocha Filho G.N., da Conceição L.R.V. (2024). Biodiesel production from waste cooking oil using an innovative magnetic solid acid catalyst based on Ni–Fe ferrite: RSM-BBD optimization approach. J. Ind. Eng. Chem..

[41] Wang Q., Zhang R., Liu M., Ma L., Zhang W. (2023). Co-Immobilization of lipases with different specificities for efficient and recyclable biodiesel production from waste oils: Optimization using response surface methodology. Int. J. Mol. Sci..

[42] Mora J.M.R., Lacson C.F.Z., Choi A.E.S., Chung T.-W., Retumban J.D., Abarca R.R.M., Grisdanurak N., de Luna M.D.G. (2024). Biodiesel production from soybean oil via LiOH-pumice catalytic transesterification and BBD-RSM optimization. Energy Rep..

[43] Buasri A., Sirikoom P., Pattane S., Buachum O., Loryuenyong V. (2023). Process optimization of biodiesel from used cooking oil in a microwave reactor: A case of machine learning and Box–Behnken design. ChemEngineering.

[44] Monteiro R.R.C., Arana-Peña S., da Rocha T.N., Miranda L.P., Berenguer-Murcia Á., Tardioli P.W., dos Santos J.C.S., Fernandez-Lafuente R. (2021). Liquid lipase preparations designed for industrial production of biodiesel. Is it really an optimal solution?. Renew. Energy.

[45] Wang X., Zhao X., Qin X., Zhao Z., Yang B., Wang Y. (2021). Immobilized MAS1 lipase-catalyzed synthesis of n-3 PUFA-rich triacylglycerols in deep eutectic solvents. J. Oleo Sci..

[46] Parandi E., Safaripour M., Mosleh N., Saidi M., Rashidi Nodeh H., Oryani B., Rezania S. (2023). Lipase enzyme immobilized over magnetic titanium graphene oxide as catalyst for biodiesel synthesis from waste cooking oil. Biomass Bioenergy.

[47] Wei H., Wang Q., Zhang R., Liu M., Zhang W. (2023). Efficient biodiesel production from waste cooking oil by fast co-immobilization of lipases from Aspergillus oryzae and Rhizomucor miehei in magnetic chitosan microcapsules. Process Biochem..

[48] Liu M., Ma L., Zhang Z., Zhang X., Zhang W. (2024). Development of a sustainable co-immobilized lipases on Co^2+^-chelated magnetic metal-organic framework for efficient biodiesel production from waste oil. Mol. Catal..

[49] Sheldon R.A., Basso A., Brady D. (2021). New frontiers in enzyme immobilisation: Robust biocatalysts for a circular bio-based economy. Chem. Soc. Rev..

